# ATP13A3 facilitates polyamine transport in human pancreatic cancer cells

**DOI:** 10.1038/s41598-022-07712-4

**Published:** 2022-03-08

**Authors:** Vandana Sekhar, Thomas Andl, Otto Phanstiel

**Affiliations:** 1 Department of Medical Education, College of Medicine, University of Central Florida, 12722 Research Parkway, Orlando, FL 32826-3227 USA; 2 Burnett School of Biomedical Sciences, University of Central Florida, 12722 Research Parkway, Orlando, FL 32826 USA

**Keywords:** Cell biology, Membrane trafficking, Cancer, Ion channels

## Abstract

The purpose of this study is to provide an increased understanding of the molecular mechanisms responsible for mammalian polyamine transport, a process that has been a long-standing ‘black box’ for the polyamine field. Here, we describe how ATP13A3, a P-type ATPase, functions as a polyamine transporter in response to different polyamine stimuli and polyamine-targeted therapies in highly proliferating pancreatic cancer cells. We assessed the expression, cellular localization and the response of the human ATP13A3 protein to polyamine treatments in different pancreatic cancer cell lines using Western blot and immunofluorescence microscopy. Using CRISPR mutagenesis and radiolabeled polyamine uptake assays, we investigated the role of ATP13A3 protein in polyamine transport. Highly metastatic cancer cells with high polyamine import express higher levels of the full-length ATP13A3 compared to cells with slow proliferation and low import activity. Highlighting its role in polyamine trafficking, the localization of ATP13A3 is altered in the presence of polyamine stimuli and polyamine-targeted therapies in these cells. Using CRISPR mutagenesis, we demonstrate that the first membrane-associated domain of this protein is critical and indispensable for its function as a spermidine and spermine transporter in cells. Further analysis of existing databases revealed that pancreatic cancer patients with high expression of ATP13A3 have decreased overall survival consistent with the role of intracellular polyamines in supporting tumor growth. Our studies shed light on the mysterious polyamine transport process in human cells and clearly establishes ATP13A3 as an intrinsic component of the spermidine and spermine transport system in humans.

## Introduction

Pancreatic ductal adenocarcinomas (PDACs) account for approximately 95% of primary pancreatic cancers, with a five-year survival rate of < 9%. (American Cancer Society Cancer Facts & Figures., 2019). These cancers have an increased addiction to the native polyamines (putrescine (PUT), spermidine (SPD) and spermine (SPM)), which are low molecular weight aliphatic amines that contribute to tumorigenesis, metastasis and immune privilege^1–4^. In normal cells, intracellular polyamine homeostasis is tightly regulated via a balance between biosynthesis, catabolism and transport^5^. However, in many cancers including PDAC, polyamine metabolism is often dysregulated with increased intracellular polyamine levels. Several chemoprevention strategies have been developed to target the high dependence of cancer cells on polyamines^6^. Combination therapies involving the polyamine biosynthesis inhibitor α-difluoromethylornithine (DFMO) and a polyamine transport inhibitor (PTI) deprive cancer cells of polyamines and have shown promising results including reduced tumor growth both in vitro and in a PDAC mouse model^7,8^. However, due to our limited understanding of the molecular mechanisms of mammalian polyamine transport, further optimization of this approach has been challenging^9^.

Very little is known about polyamine uptake and only a few proteins have been suggested as polyamine transporters^10^. For example, ATP13A2 has been shown to be a lysosomal polyamine exporter^11^, and SLC12A8 was originally suggested as another polyamine importer, but is now designated as an amino-acid and a nicotinamide mononucleotide transporter^12,13^. Recently, ATP13A3 has been suggested as a major mammalian polyamine transporter as demonstrated in Chinese hamster ovary cells^14^. Our previous study showed that siRNA knockdown of ATP13A3, a member of the P5-subfamily of the P-type ATPases, significantly decreased growth of DFMO treated human pancreatic cancer cells^15^. Although, reports implicate *ATP13A3* gene mutations in the pathogenicity of pulmonary arterial hypertension (PAH) and its altered expression has been reported in cervical and pancreatic cancers, its regulation and molecular characteristics are still poorly elucidated^16–19^. Here, we define the important role of ATP13A3 in polyamine transport in human cells using a panel of pancreatic cancer cells that differ in their ability to import polyamines. Different pancreatic cancer cell lines demonstrate preferential expression of the ATP13A3 protein and exhibit distinct cytosolic and plasma membrane localization patterns in the presence of polyamine stimuli and polyamine-targeted therapies. Importantly, CRISPR mutagenesis revealed that the first membrane-associated domain of the ATP13A3 protein is critical for its function as a polyamine transporter, specifically for spermidine and spermine import into cells. This discovery provides an important first step in understanding the role of proteins involved in the polyamine transport pathway and provides a defined target for combination therapies that seek to starve cancers of their polyamine pro-growth factors^9^.

## Results

### Distinct expression and localization of the ATP13A3 protein in pancreatic cancer cell lines

**Figure 1:**
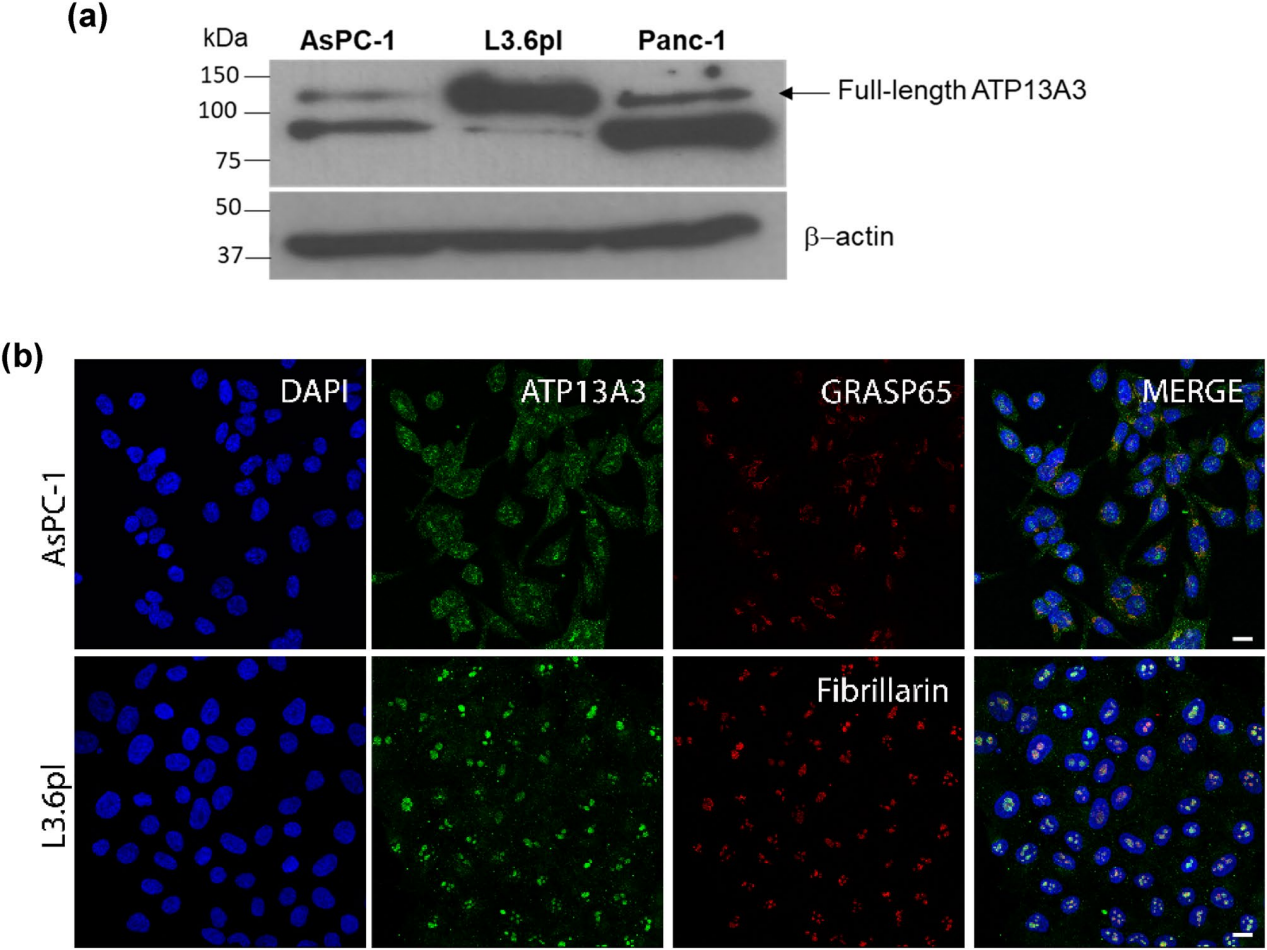
Distinct expression and localization patterns of the ATP13A3 protein in pancreatic cancer cell lines. (**a**) Full-length ATP13A3 protein expression in the three cell lines. β-actin was used as a loading control. (**b**) Cellular localization of the ATP13A3 protein in AsPC-1 cells (top) and L3.6pl cells (bottom) with DAPI stained nuclei in blue, ATP13A3 protein in green, Golgi and nucleolar marker in red. (Scale bar, 10 µm). The original blots are presented in Supplementary Fig. SI.8.

Our prior study showed that different human pancreatic cancer cell lines exhibited different rates of radiolabeled SPD (^3^H-SPD) uptake under DFMO pressure and characterized these cell lines, L3.6pl, Panc1, and AsPC-1 cells as high, medium, and low polyamine importers, respectively^15^. Since, the study suggested a relationship between ATP13A3 expression and polyamine transport activity, here we compared the expression and localization of the ATP13A3 protein in these cell lines. The L3.6pl cells (high polyamine importer) showed high expression of the full-length ATP13A3 protein (~ 130 kDa). In contrast, Panc-1 (medium importer) and AsPC-1 (low importer) cells had, respectively, lower expression of the full length ATP13A3 protein (Fig. 1a). We also observed an additional band at ~ 78 kDa potentially representing a truncated isoform of ATP13A3 as has been reported by Habtemichael et al.^20^.

Further studies focused on the two pancreatic cancer cell lines that dramatically differed in their ability to import polyamines (L3.6pl vs AsPC-1). Different subcellular localization of ATP13A3 was observed in these cells. The protein mainly localized in the nucleolus and the cytoplasm of L3.6pl cells. Using fibrillarin as a marker for nucleolar protein, we observed that ATP13A3 closely localized to fibrillarin and importantly, a fraction of the protein was also observed along the plasma membrane. In contrast, in AsPC-1 cells, ATP13A3 mainly localized in the cytoplasm, specifically to the Golgi apparatus as confirmed by co-localization with the Golgi marker, GRASP65 and to a lesser extent along the plasma membrane (Fig. 1b).

### Altered localization of the ATP13A3 protein in response to polyamine stimuli and polyamine-targeted therapies in cells with high import activity

**Figure 2:**
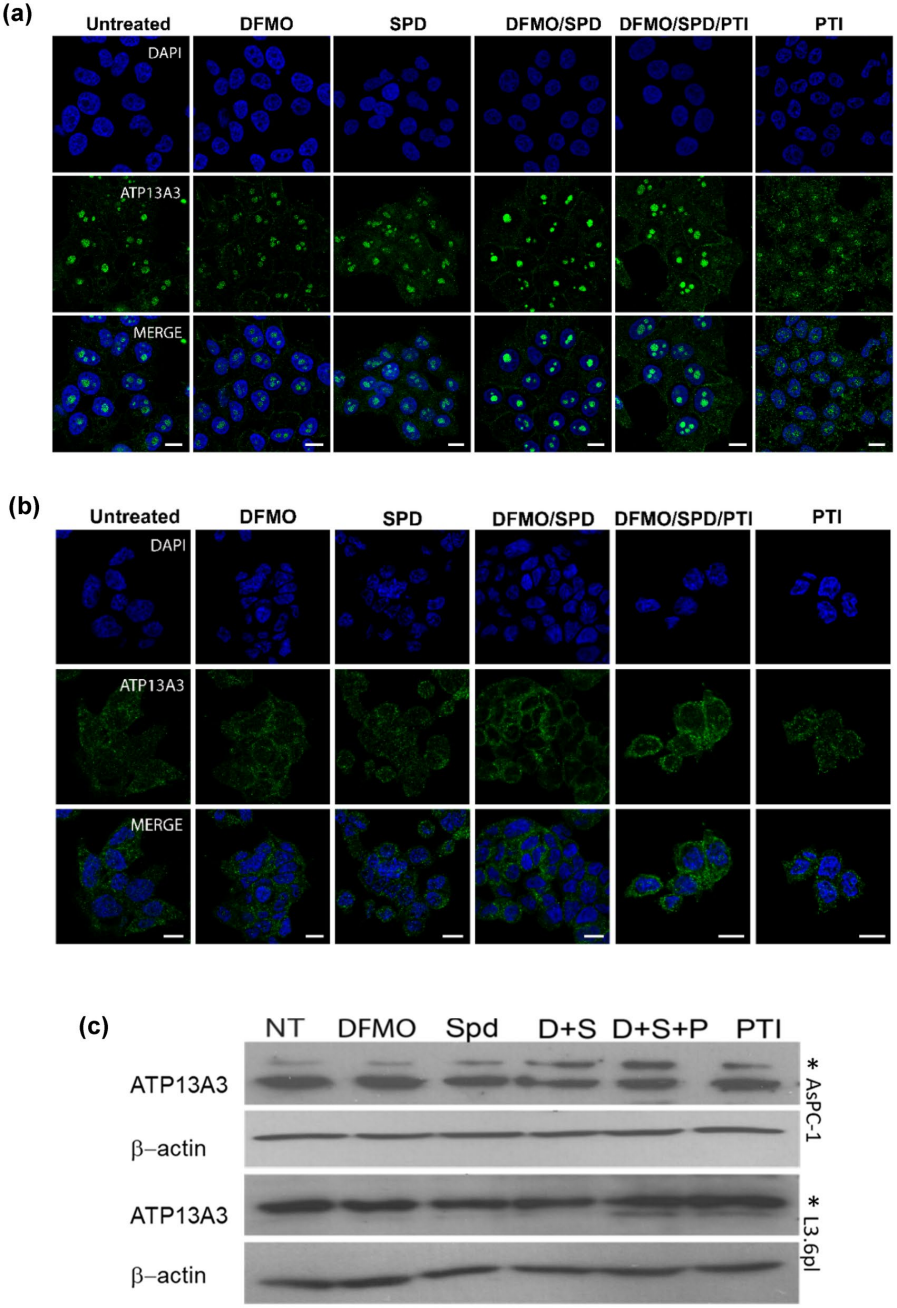

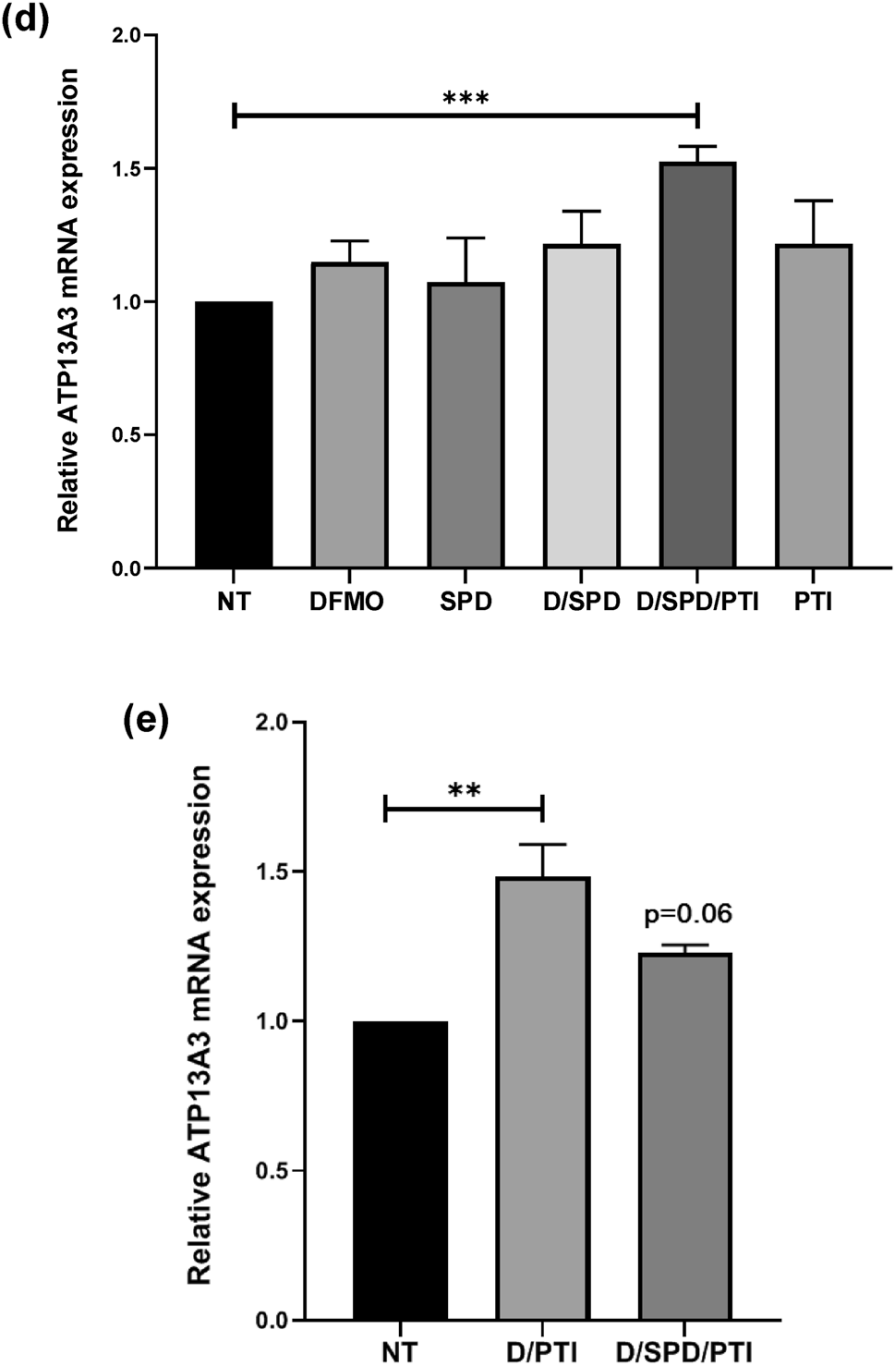
Effects of polyamine stimuli on the localization and the expression levels of the ATP13A3 protein in pancreatic cancer cell lines. Localization of ATP13A3 (green) following treatment with different polyamine stimuli in L3.6pl cells (**a**) and AsPC-1 cells (**b**). Cells were treated with DFMO (11.8 mM for AsPC-1 and 4.2 mM for L3.6pl), 1 µM SPD, a combination of DFMO and SPD (D + Spd), PTI, trimer44NMe (4 µM) or a combination of DFMO + Spd + PTI (Scale bar, 10 µm). (**c**) Expression of the full length ATP13A3 protein (marked with an *) in L3.6pl and AsPC-1 cells treated with DFMO (D), spermidine (Spd or S), DFMO + Spd, DFMO + Spd + PTI or PTI only with β-actin as internal control. The cell lysates of treated L3.6pl cells and AsPC-1 cells were loaded on separate SDS gels. Respective internal controls were run in parallel as the ATP13A3 protein for each of the cell lines. Relative ATP13A3 mRNA transcripts measured by qRT-PCR in AsPC-1 cells treated with DFMO, SPD, DFMO + SPD, DFMO + SPD + PTI or PTI only (**d**) or D + PTI and D + SPD + PTI (**e**). The data are representative of mean ± SD of three independent experiments (***P = 0.0005 and **P = 0.008). Note: The D/SPD/PTI entry in (**e**) was very close to also being statistically significant (as it reached the 94% confidence limit) when compared to the untreated NT control. The original blots are presented in Supplementary Fig. SI.8.

We next investigated the relative responses of the two cell lines to polyamine stimuli and polyamine-targeted therapies. Specifically, the cells were treated with DFMO, SPD or a combination of DFMO, SPD, and/or the PTI trimer44NMe for 72 h. DFMO treatment is known to increase polyamine uptake because cells respond to inhibition of polyamine biosynthesis via increased import^15,21^. We noted that DFMO treatment resulted in an increased localization of ATP13A3 along the plasma membrane of L3.6pl cells. However, treatment with SPD alone/or SPD in the presence of DFMO each resulted in a loss or a reduction of plasma membrane bound ATP13A3, demonstrating instead an increased diffused cytoplasmic staining presumably due to internalization of ATP13A3 and SPD (Fig. 2a). Notably, upon induction of intracellular polyamine stress (DFMO + SPD + PTI), the membrane bound localization pattern for ATP13A3 reappeared more distinctly. In contrast, treatment of AsPC-1 cells with DFMO, SPD, PTI independently did not result in significant changes in the localization of the protein, supporting the idea that these cells rely more heavily on polyamine biosynthesis for their proliferation (Fig. 2b). Our findings that the 72 h DFMO IC_50_ for AsPC-1 cells is 11.8 mM, which is significantly higher than the 72 h IC_50_ of 4.2 mM for L3.6pl cells and that these cells were not rescuable with exogenous SPD under DFMO pressure, further provide evidence of their low polyamine import activity and high dependence on biosynthesis^15^. However, upon induction of extreme polyamine stress (DFMO + SPD + PTI), an increased localization of ATP13A3 along the plasma membrane was noted (Fig. 2b). These observations demonstrate that highly metastatic cancer cells (L3.6pl) and slow proliferating cells (AsPC-1) to a lesser extent are sensitive to different polyamine stimuli and that the ATP13A3 protein in these cells responds to the different stimuli by altering its cellular localization. Putting these observations together, the fact that untreated L3.6pl and AsPC1 cells have different expression levels of full length ATP13A3 protein and different ATP13A3 localization patterns provides a tantalizing rationale for their different polyamine uptake activity.

### Effects of polyamine stimuli on ATP13A3 protein levels

Given that there were dramatic changes in the localization of ATP13A3 with polyamine stimuli/therapy, we next examined their effects on the overall protein levels. As shown previously in Fig. 1a, L3.6pl and AsPC1 cells have high and low full length ATP13A3 expression, respectively. In L3.6pl cells, there were no significant changes detected in the full-length ATP13A3 protein levels in the presence of the different polyamine targeted stimuli (Fig. 2c). In contrast, AsPC-1 cells showed an increase in the levels of the full-length protein under conditions of DFMO + SPD and DFMO + SPD + PTI stimuli (Fig. 2c and Fig SI 1). The increase in protein expression also correlated with a corresponding increase at the mRNA level with a significant increase observed in cells under intracellular polyamine stress (DFMO + SPD + PTI) (Fig. 2d). This increase in expression mirrored the increased localization of the ATP13A3 protein along the membrane in AsPC-1 cells under intracellular polyamine stress. However, treatment of AsPC-1 cells with only DFMO + PTI revealed that SPD was not critical for this response since, the stress generated by both inhibitors was enough to trigger the increase in the ATP13A3 mRNA expression (Fig. 2e). These data together indicate that a complete blockade of polyamine biosynthesis and uptake triggers cells like AsPC-1 (with low basal levels of the full-length protein and high dependence on biosynthesis) to start making more full-length ATP13A3 to upregulate polyamine import.

### SLC12A8 does not play a role in polyamine transport in pancreatic cancers

A recently described nicotinamide mononucleotide transporter, SLC12A8, has been suggested as a putative polyamine-amino acid transporter and recurrent noncoding regulatory mutations in this gene have been implicated in PDAC^12,13,22^. To investigate whether SLC12A8 plays any role in polyamine transport, we first examined the expression and localization of the protein in the different pancreatic cancer cells. Like ATP13A3, the SLC12A8 protein is also expressed as different isoforms (ranging from 38-78KDa) produced by alternative splicing. Higher expression of the full-length protein was observed in AsPC-1 cells with lower molecular weight bands potentially corresponding to the other isoforms also seen in all cell types (Fig. SI 2a). Again, the two cell lines that represent extremes of polyamine transport activity, L3.6pl and AsPC-1 cells, were chosen to examine the cellular localization of the SLC12A8 protein. A cytoplasmic distribution of the protein with a more distinct membranous localization was seen in AsPC-1 cells (Fig. SI 2b). In L3.6pl cells, though mostly cytoplasmic, only a fraction of the protein localized along the membrane. Notably, no significant alteration was observed in the localization of the SLC12A8 protein in either L3.6pl or AsPC-1 cells in response to the different polyamine treatments. In L3.6pl cells, the protein seemed to be primarily cytoplasmic in the presence of DFMO and/or SPD (Fig. SI 2c), whereas in AsPC-1 cells, it localized along the membrane as seen in untreated cells (Fig. SI 2d). Thus, unlike ATP13A3 in L3.6pl cells, SLC12A8 did not appear to respond to polyamine stimuli by altering its cellular localization in these cell lines.

### Cells with functionally defective ATP13A3 demonstrate an impaired SPD and SPM uptake

**Figure 3:**
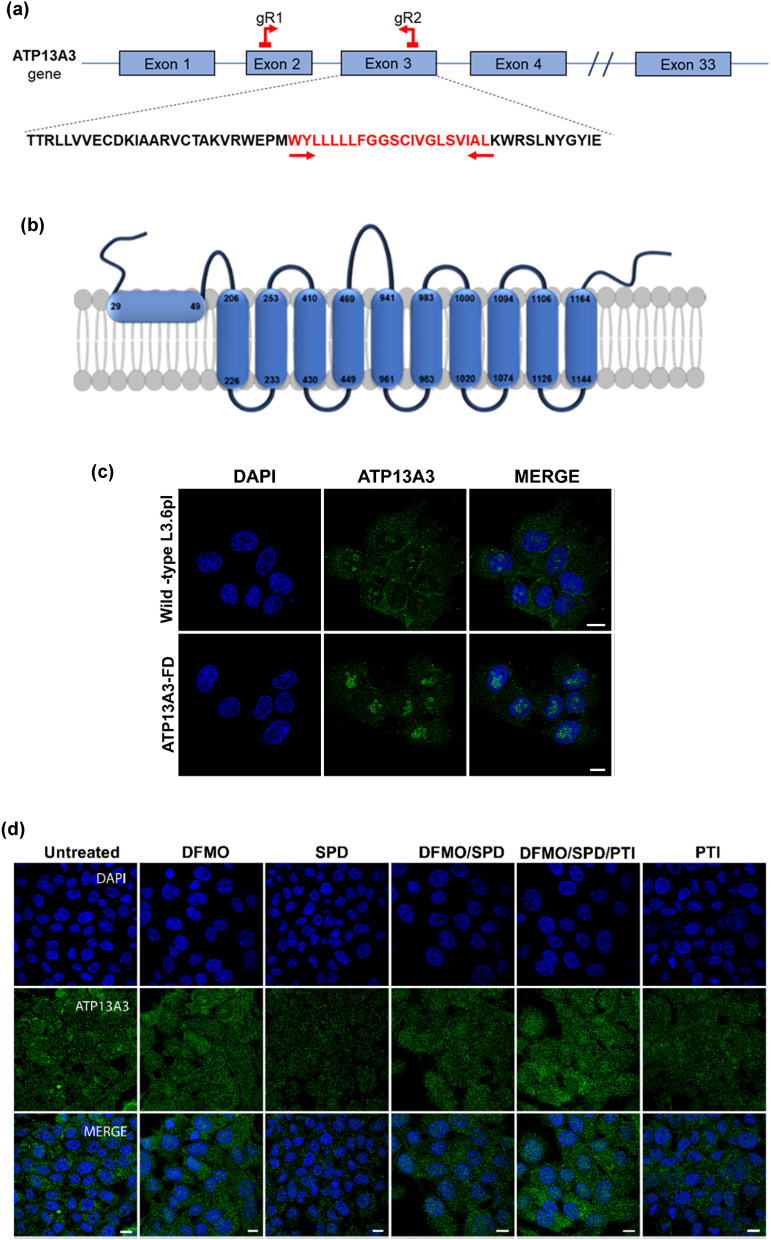
CRISPR mutagenesis results in a modified ATP13A3 protein lacking a membrane associated domain. (**a**) Schematic diagram showing the region of the ATP13A3 gene targeted for the CRISPR mutagenesis study. Two guide RNAs (gR1 and gR2) targeting exon 2 and exon 3, respectively are shown. The amino acids spanning the first transmembrane domain within the exon 3 are shown in red. (**b**) Based on homology modeling, a schematic representation of the ATP13A3 transmembrane domains along with the deleted first membrane associated region in the ATP13A3Δ(29–49) protein. (**c**) Immunofluorescence images showing the localization of the ATP13A3 protein (green) in wild-type L3.6pl cells (upper panel) and the ATP13A3-FD cells (lower panel) treated with DFMO at 4.2 mM for 72 h. DAPI stained nuclei are in blue. (**d**) Confocal microscopy images showing the cytoplasmic localization of the defective ATP13A3Δ(29–49) (green) following different polyamine stimuli or treatment with DFMO (D), spermidine (Spd), DFMO/Spd, DFMO/Spd/PTI or PTI in ATP13A3-FD cells. DAPI stained nuclei are in blue. (Scale bar, 10 µm).

To further investigate the functionality of the *ATP13A3* gene in cellular response to polyamine targeting therapies, we performed CRISPR-Cas9 mutagenesis. Specifically, guide RNAs targeting exon 2 and exon 3 of the *ATP13A3* gene were designed (Fig. 3a and SI 3a). DNA sequencing from the resulting ATP13A3 CRISPR clone, revealed that the gRNA mediated deletion resulted in a loss of more than half of exon 2 and 3, in addition to the intronic region. Precisely, the clonal cells had a deletion of 153 bp resulting in the loss of 51 amino-acids but still produced an in-frame nearly full-length ATP13A3 protein (Fig. SI 3a). Importantly, based on homology modeling, the deletion included a region that encodes the first membrane-associated domain of the protein (UniProtKB Q9H7F0; amino acid 29–49) (Fig. 3b).

Thus, the ATP13A3 mutant cell line (denoted as ATP13A3-FD, functionally dead) expresses a mutated ATP13A3 that is missing the first membrane associated domain (ATP13A3Δ(29–49)). Unlike the wild-type L3.6pl cells, the ATP13A3-FD cells demonstrated a complete loss of the membranous localization of the ATP13A3 protein when cells were treated with DFMO for 72 h. In the ATP13A3-FD cells, the ATP13A3Δ(29–49) protein appeared in the nucleolus and the cytoplasm, with no localization along the cell membrane (Fig. 3c). Furthermore, the localization of the ATP13A3Δ(29–49) protein was not altered in response to any of the polyamine stimuli, namely, DFMO, SPD, PTI, independently or in combination, indicating that the ATP13A3Δ(29–49) protein is functionally defective in its response to polyamine stimuli (Fig. 3d). Interestingly, HPLC analysis revealed a reduction in the SPD levels in the ATP13A3-FD cells in addition to a reduction in the total polyamine levels compared to wild-type cells (Fig. SI 4). This is likely due to the inability of the ATP13A3-FD cells to import exogenous SPD.

**Figure 4:**
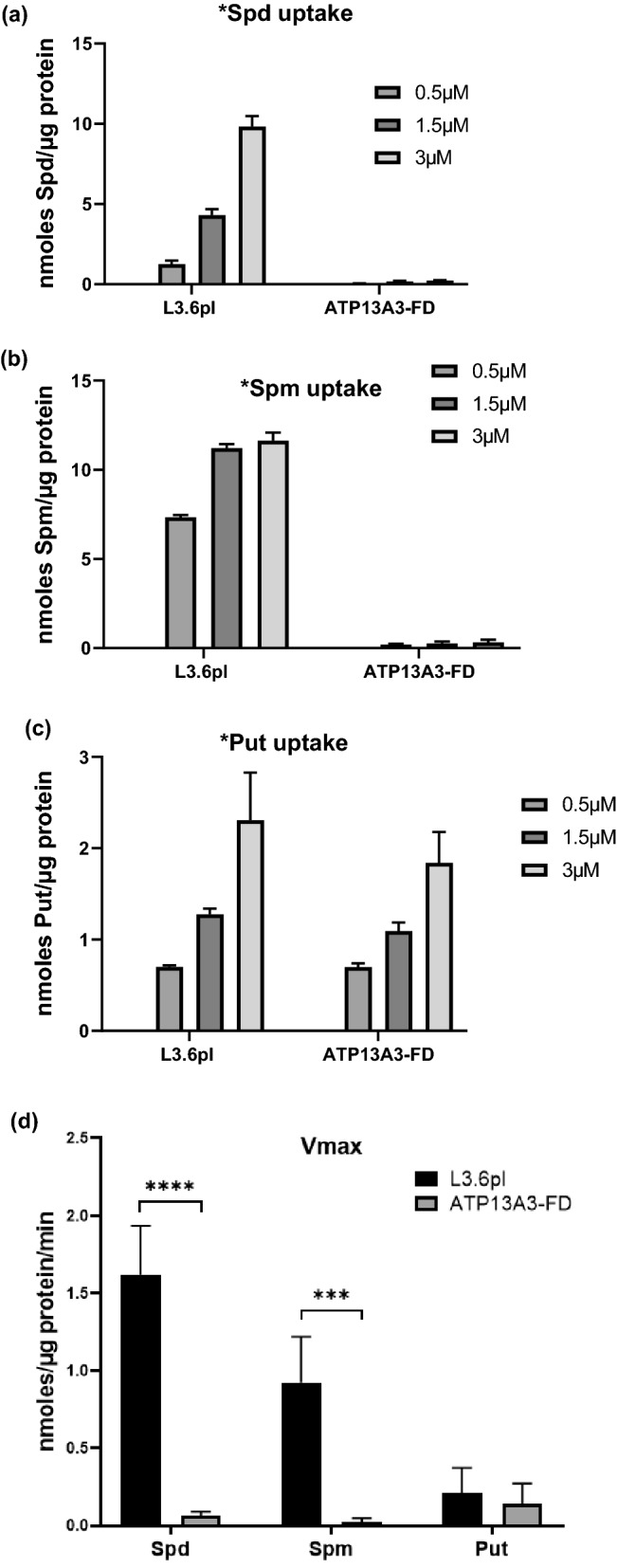

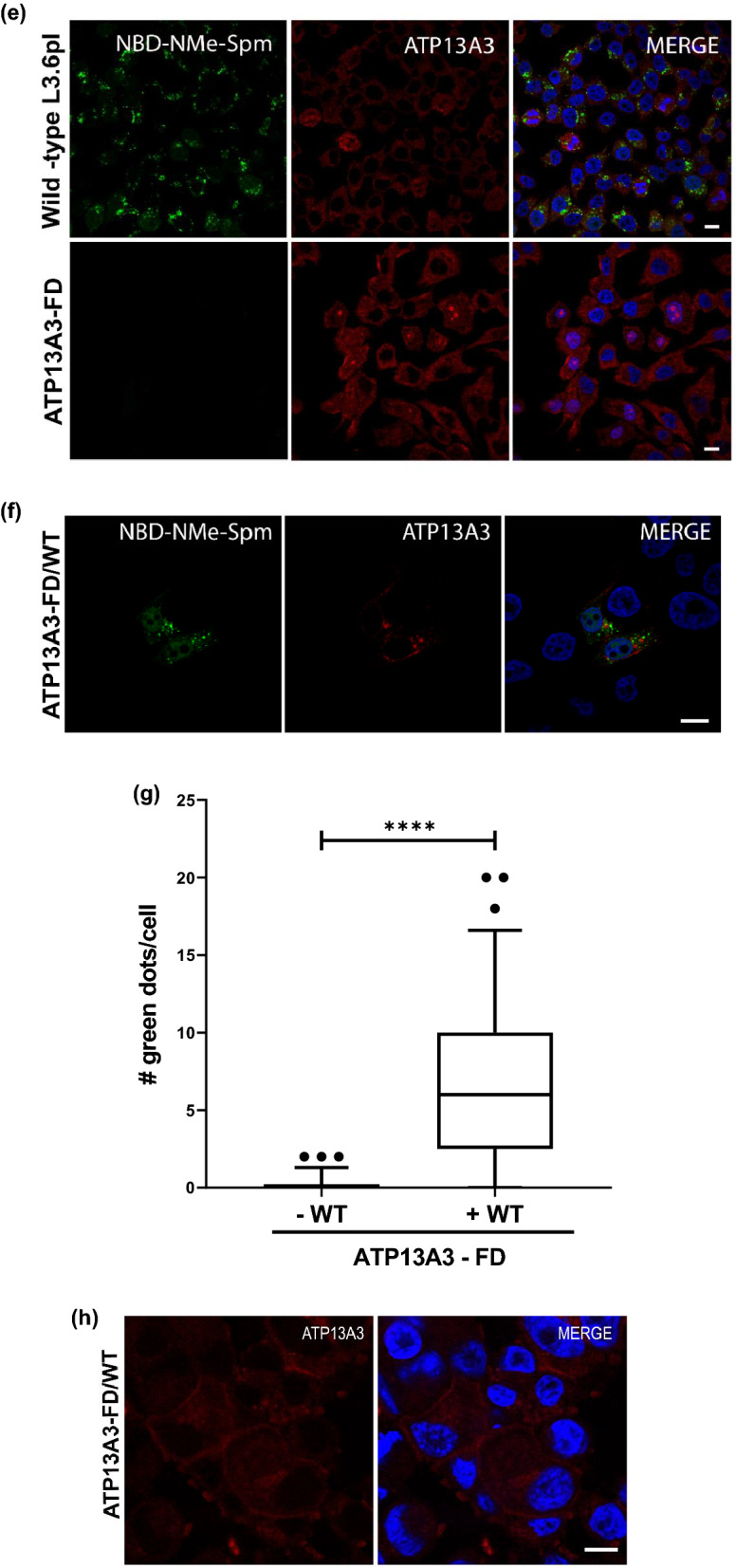
Cells with functionally defective ATP13A3 demonstrate impaired spermidine and spermine uptake. Graphical representation showing the amount of exogenous 3H-Spd (**a**),14C-Spm (**b**) and 3H-Put (**c**) uptake by wild-type L3.6pl cells and ATP13A3-FD cells after cells were dosed with different concentrations of 3H-Spd,14C-Spm or 3H-Put (0.5–3 µM) for 15 min. (**d**) Graphical representation of the rate of 3H-Spd, 14C-Spm and 3H-Put uptake per min (Vmax) expressed as nmoles/µg of protein/min in wild type L3.6pl and ATP13A3-FD cells. ****p < 0.0001, ***p = 0.0003. (**e**) Confocal microscopy images showing the intracellular uptake of the green fluorescent probe NBD-NMe-Spm (1 µM) 4 h post treatment in the wildtype L3.6pl and ATP13A3-FD. The ATP13A3 protein is shown in red and the DAPI stained nuclei are shown in blue in the merged image (Scale bar, 10 µm). (**f**) Representative immunofluorescence image showing the uptake of the green fluorescent probe NBD-NMe-Spm (1 µM) 4 h post treatment in ATP13A3-FD/WT (red) rescue cells. In red the overexpressed ATP13A3-FusionRed protein is shown (Scale bar, 10 µm). (**g**) Quantification of the number of green spots observed in ATP13A3-FD/ + WT (rescue) and ATP13A3/-WT (control) cells (****P < 0.0001). (h) Re-localization of the overexpressed ATP13A3 along the plasma membrane in ATP13A3-FD/WT (red) rescue cells under DFMO pressure.

To further elucidate how the functional loss of ATP13A3 specifically affected the cells ability to import polyamines, we compared the rate of radiolabeled polyamine uptake in the wild-type and ATP13A3-FD cells in a span of 15 min. Dosing the cells with increasing concentration of radiolabeled SPD and SPM ranging from 0 µM to 3 µM resulted in a corresponding increase in the intracellular radioactivity in wild-type cells. On the contrary in the ATP13A3-FD cells, even at the highest doses of exogenous radiolabeled SPD or SPM, very little intracellular radioactivity was detected (Fig. 4a, b). Interestingly, comparison of the radiolabeled PUT uptake between the two cell lines demonstrated no significant difference in the levels of intracellular radiolabeled PUT, indicating that PUT uptake in L3.6pl relies on a transporter other than ATP13A3 (Fig. 4c). Furthermore, the lack of SPD and SPM uptake was reflected in the significantly reduced rate of SPD and SPM uptake per min (Vmax of 0.06 ± 0.02 and 0.02 ± 0.02 nmol/µg protein/min, respectively) observed for the ATP13A3-FD cells compared to the wild-type cells (Vmax 1.60 ± 0.31 and 0.90 ± 0.29 nmol/µg protein/min, respectively) (Fig. 4d).

To further confirm that the ATP13A3 protein is essential for SPD transport, we compared the uptake of a fluorescently labeled SPM probe, a 4-nitro-benzo- [1,2,5] oxadiazolyl spermine derivative (NBD-NMe-SPM) in wild-type and mutant cells. Since one end of SPM is capped with the NBD fluorophore, the probe presents a SPD tail for cells to import. The wild-type cells demonstrated a significant uptake of the green fluorescent probe following treatment with 1 µM NBD-NMe-SPM for 4 h, whereas the ATP13A3-FD cells were defective in their uptake of the probe (Fig. 4e). Interestingly, expression of the wild-type functional ATP13A3 in the FD background (denoted FD/WT) for rescue, resulted in significant uptake of the NBD-NMe-SPM probe in the rescued cells (Fig. 4f, g). Only cells expressing the wild-type ATP13A3 (red) demonstrated the uptake of the green fluorescent probe, further confirming the critical role of ATP13A3 in polyamine transport (Fig. 4g). Additionally, we also observed the re-localization of the overexpressed wild-type ATP13A3 (red) along the plasma membrane in the ATP13A3-FD/WT rescued cells under DFMO pressure (Fig. 4h).

We also determined the sensitivity of the ATP13A3-FD cells to DFMO by dosing the cells with an increasing concentration of DFMO (0.01–10 mM) for 72 h and then measuring cell growth using the MTS assay. As expected, the ATP13A3-FD cells with virtually no SPD or SPM import were highly sensitive to DFMO treatment. The 72 h DFMO IC_50_ value of the mutant cells was found to be 0.1 mM (Fig. SI 5), in contrast, to previously reported 4.2 mM for wild-type cells^15^. In this regard, the ATP13A3-FD cells were 42 times more sensitive to DFMO than the wild type cells. In addition, unlike the wild-type cells, the addition of exogenous SPD (1 µM), in the presence of DFMO, did not rescue the growth of the ATP13A3-FD cells. In sum, these observations are all consistent with the inability of the ATP13A3-FD cells to import SPD and SPM to replenish their polyamine pools resulting in high sensitivity to DFMO. Taken together our studies show that ATP13A3 is involved in transport of SPD and SPM, but not PUT, indicating that different polyamines use different transporters to enter cells^23^.

Additionally, the ATP13A3-FD cells allowed us to test the hypothesis that the two proteins (ATP13A3 and SLC12A8) may compensate for each other. To test if there was any upregulation of SLC12A8 protein levels in the ATP13A3-FD cell line, we compared the levels of SLC12A8 protein in the wild type and the ATP13A3-FD cells. There was no significant difference in the levels of any of the different isoforms of the SLC12A8 protein between the wild-type and the ATP13A3-FD cells, suggesting the two proteins do not compensate for each other (Fig. SI 6). This is further supported by the observation that the Vmax for ^3^H-SPD and ^14^C-SPM uptake was very low for the ATP13A3-FD cells consistent with an inability of SLC12A8 to rescue polyamine transport activity, when ATP13A3 function was impaired.

### Clinical significance of ATP13A3

**Figure 5:**
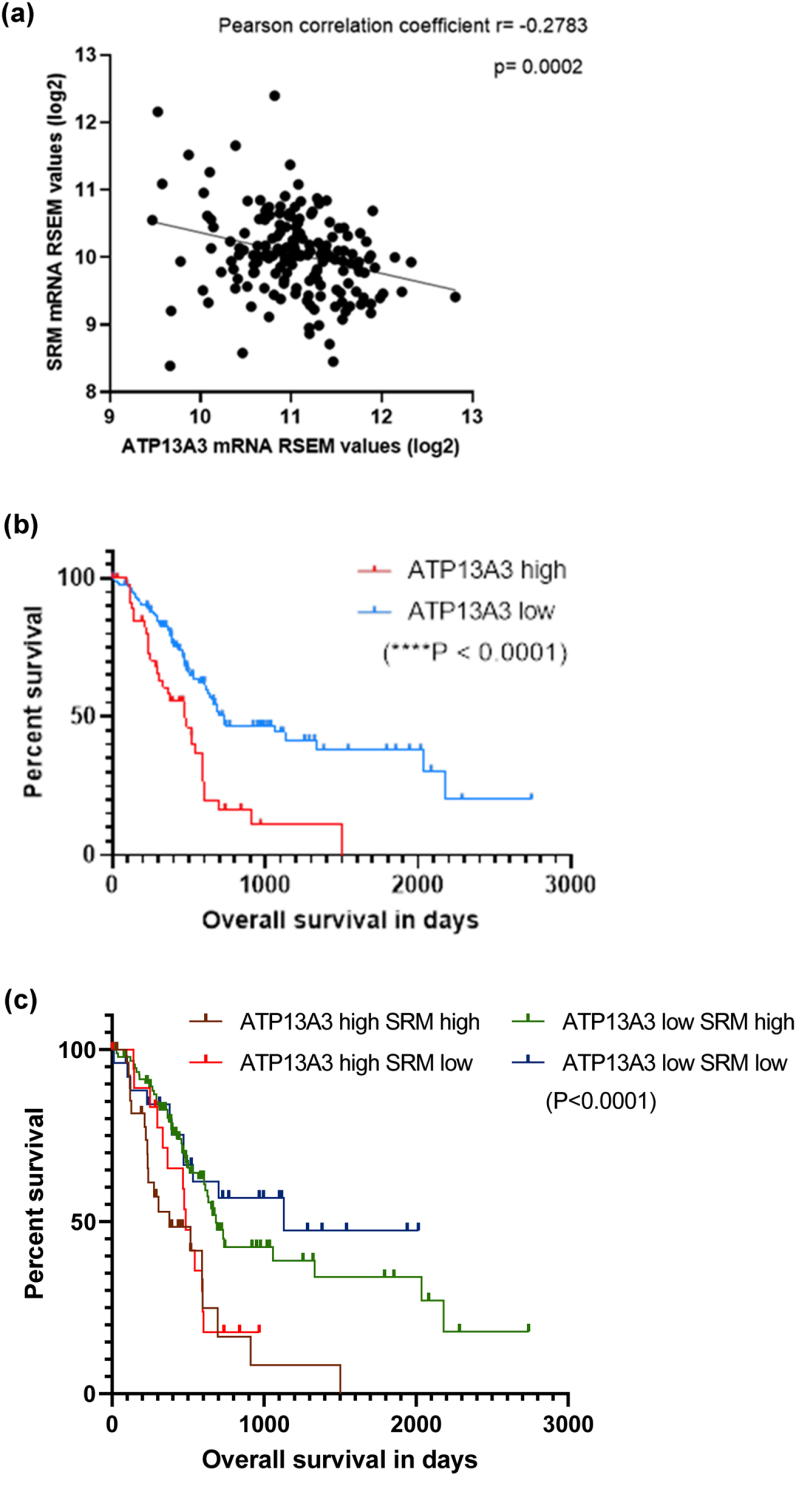
Clinical significance of ATP13A3 indicating high ATP13A3 expression is unfavorable for pancreatic cancer patient survival. (**a**) Graph representing the inverse correlation of SRM mRNA expression with ATP13A3 mRNA expression, obtained using the Pancreatic adenocarcinoma (TCGA, PanCancer Atlas) dataset. Pearson correlation coefficient was used to express the level of correlation. Correlation of ATP13A3 (**b**) and ATP13A3-SRM (**c**) expression with pancreatic cancer patient survival, using the “GDC TCGA Pancreatic Cancer” dataset in the UCSC Xena Browser. Statistical significance was determined using the Log-rank (Mantel-Cox) test.

Our *in-vitro* findings establish ATP13A3 as the major importer of both spermidine and spermine in human pancreatic cells. Predictably, analysis of the correlation of polyamine metabolic genes with ATP13A3 mRNA expression showed an inverse correlation with many of the polyamine biosynthesis genes such as spermidine synthase (SRM) in PDACs analyzed in the cBiO Cancer Genomics dataset (Fig. 5a and SI 7a-b). However, genes such as the pro-proliferation genes (CDK6) showed a positive association with ATP13A3 mRNA expression (Fig. SI 7c). Importantly, high ATP13A3 expression in tumors was associated with poor prognosis and decreased patient survival compared to low expression (Fig. 5b). Additionally, we also observed that the ATP13A3-negatively associated genes tend to be favorably associated with survival (Fig. 5c). This further confirmed our observation in PDAC patient samples that tumors exhibit some level of heterogeneity in the expression of ATP13A3 with some PDACs expressing higher ATP13A3 than others (Fig. SI 7d-e). These findings underscore the importance of understanding how ATP13A3 affects patient survival independent of the polyamine biosynthesis genes.

## Discussion

Our studies reveal a mechanistic understanding of how ATP13A3 functions in polyamine transport in mammalian cells. Highly metastatic cancer cells expressing high levels of the full-length ATP13A3 protein are sensitive to polyamine targeted therapies and respond to polyamine stimuli by altering the cellular localization of ATP13A3. DFMO treatment of rapidly-proliferating L3.6pl cells significantly increases the membranous localization of ATP13A3. However, in the presence of exogenous SPD, the membrane localization is significantly reduced, resulting instead, in a diffuse cytoplasmic distribution. We speculate that when cells are starved of polyamines, ATP13A3 is likely at the membrane ready to import exogenous SPD that may become available. Once the SPD is taken up, ATP13A3 is internalized into vesicles that transport SPD intracellularly. In support of this premise, ATP13A3 has been shown to colocalize with certain recycling endosomes such as Rab11 and to a lesser extent to late endo-/lysosomes in an overexpression system^24^. Future studies could shed light on the role of endosomes and ATP13A3 in trafficking polyamines between the cytosol and the plasma membrane.

Our CRISPR mutagenesis studies reveal that the first membrane-associated domain encoded by the *ATP13A3* gene is critical for its function as a polyamine transporter, specifically for SPD and SPM import. This highlights the likely presence of another transporter for PUT as previously suggested^23^. Deletion of this key domain dramatically alters the ability of ATP13A3 to respond to polyamine stimuli as demonstrated by the failure of ATP13A3Δ(29–49) protein to exhibit membranous localization under conditions of polyamine starvation in the ATP13A3-FD cells. Interestingly, a hydrophobic stretch of twenty-three amino acids in the N-terminus of the related ATP13A2 protein is critical for its binding to the cytosolic membrane surface of the lysosomes via interactions with the phosphatic acid and phosphatidylinositol (3,5) bisphosphate present in the membranes^25^. Based on homology modeling, the first membrane-associated domain of ATP13A3 corresponds to this hydrophobic stretch of the ATP13A2 thereby explaining its importance in the membranous localization of the protein. Furthermore, the mutant cells lacking this critical region appear to be highly sensitive to DFMO (42 times more sensitive than wild-type), due to their inability to transport SPM and SPD.

Our findings reveal that the rapidly dividing L3.6pl cells with high expression of ATP13A3 exhibit high Vmax for SPD and SPM and thus, are high polyamine transporters to meet their high demand for polyamine growth factors during rapid proliferation^15^. Additionally, ATP13A3 expression has been found to be relatively high in mice during early embryonic development and siRNA knockdown of ATP13A3 results in reduced proliferation of endothelial cells, further linking ATP13A3 expression with proliferation^24,26^. In contrast, cells with slow proliferation rates, namely AsPC-1 cells, express lower amounts of the full-length ATP13A3 protein (and higher levels of truncated protein) and demonstrate a cytoplasmic localization especially within the Golgi vesicles. The ASPC-1 cells have a high dependence on polyamine biosynthesis as is evidenced by their high DFMO IC_50_ value. Interestingly, a truncated version of the ATP13A3 has been described in senescent human parenchymal kidney cells further supporting our finding that slow-growing cells express the truncated version of the protein^20^. Taken together our findings for the first time clearly demonstrate the region of the ATP13A3 protein that is crucial for its role as an SPD and SPM importer when cells are starved of polyamines and provides a well-defined drug target for future therapies that target polyamine uptake.

In summary, this is the first report to demonstrate by knock out and knock in experiments the function of a human polyamine importer and provides ATP13A3 as a starting protein target to help deconvolute the mysterious human polyamine transport system.

## Materials and methods

### Cell culture

L3.6pl pancreatic tumor cells were a gift from Dr. Isaiah Fidler (MD Anderson Cancer Center, Houston, TX). AsPC-1 and Panc-1 cell lines were obtained from ATCC. All cell lines were cultured at 37 °C in 5% CO_2_ atmosphere in RPMI-1640 media supplemented with 10% fetal bovine serum (FBS) and 1% penicillin/streptomycin.

### Reagents

DFMO was kindly provided by Dr. Patrick Woster (Medical University of South Carolina, Charleston, SC). The trimer44NMe polyamine transport inhibitor (PTI) and the N^1^-(4-nitro-benzo-[1,2,5] -oxadiazolyl) spermine derivative were synthesized in house^8,27^. Aminoguanidine was purchased from Sigma-Aldrich. Spermidine was acquired from Acros Organics and ^3^H-Spd and ^3^H-Put were purchased from Perkin-Elmer Inc. ^14^C-Spm was obtained as a gift from Dr. Tracy Murray-Stewart (Johns Hopkins). Primary antibodies for ATP13A3 (HPA029471) and β-actin were obtained from Sigma. Fibrillarin and GRASP65 antibodies were obtained from Cell Signaling Technology, and the anti-SLC12A8 antibody was custom synthesized by GenScript. Anti-mouse and anti-rabbit secondary antibodies for immunoblotting were purchased from Santa Cruz Biotechnology and anti-mouse IgG Alexa568 and anti-rabbit IgG Alexa488 were obtained from Invitrogen.

### Immunoblotting

Cell extracts were prepared in modified radioimmunoprecipitation assay (RIPA) buffer (20 mM HEPES, pH 7.0, 150 mM NaCl, 1 mM EDTA, 1% NP-40, 1% deoxycholate, 0.1% sodium dodecyl sulfate [SDS]) containing complete protease inhibitor cocktail (Roche) and PhosSTOP phosphatase inhibitor cocktail (Roche). Protein concentrations were determined using the bicinchoninic acid (BCA) protein assay kit (Pierce) according to the manufacturer’s instructions. For each sample, 50 μg of protein was separated by SDS–polyacrylamide gel electrophoresis (PAGE) and electro-transferred onto nitrocellulose membranes (BioRad). Proteins were detected with different primary antibodies followed by horseradish peroxidase-conjugated secondary antibodies. Proteins were detected on the membrane with the chemiluminescent reagent SuperSignal West Dura (ThermoScientific). ImageJ software was used to perform densitometric analysis of the blots.

### RNA preparation and quantitative reverse transcription-PCR

Total RNA was extracted using RNeasy Mini Kit (Qiagen) according to the manufacturer’s protocol. cDNA was prepared from 500 ng of RNA using QuantiTect Rev. Transcription Kit (Qiagen). Quantitative PCR reactions were set up in MicroAmp optical 96-well reaction plates (Applied Biosystems). QuantiTect SYBR Green RT-PCR Kit was used for amplifying 4 µL of the cDNA with custom primers for β-actin from Qiagen and ATP13A3 primer pairs- forward 5’-GCCATAGTGTTAGAACAGGATTTAG-3’ and reverse 5’-AACTGCCACAAGACATAGTAGAA-3’ (Integrated DNA technology, IDT). Reactions were amplified using the QuantStudio7Flex instrument. The relative abundance of ATP13A3 transcript for different conditions were compared using the equation 2^–(∆∆Ct)^ and fold expression determined relative to the no treatment (NT) control.

### Immunofluorescence assay

Cells were seeded on cover slips in 24 well plates at a concentration of 40,000–50,000 cells with 250 μM aminoguanidine in 500 μL of media per well. Before fixation, the media was removed followed by three 1× PBS washes (5 min). Cells were then fixed with 4% PFA for 20 min at room temperature. Following washes with PBS, the cells were permeabilized with 0.1% Triton X-100 in PBS for 15 min at room temperature and then blocked with 10% goat serum in PBS-T (0.1% Tween-20 in PBS) for 30 min at room temperature. Cells were then stained with primary antibody in blocking solution at 4 °C overnight in a humidified chamber followed by secondary antibody for 1 h at room temperature. Coverslips were mounted onto slides using a drop of mounting media with DAPI (Southern Biotech). Cells were imaged from randomly selected fields with a Leica SP5 confocal microscope (Leica Microsystems Inc, Buffalo Grove, IL, USA) using the 100× objective. The images were acquired using the Leica LAS AF software. Representative images are shown, and the white scale bar applies to each slide and all images were taken at the same magnification.

### CRISPR-Cas9 mediated ATP13A3 mutagenesis

Two guide RNA sequences (gRNA) targeting the *ATP13A3* gene, in the region of exon 2 and exon 3, respectively, were designed by considering their high on-target and low off target efficiency using the http://crispor.tefor.net/crispor.py program. The selected gRNA sequences AATCCACTGTAGGTATGGAC and GTCGCTTTCACCCGCCACTC were obtained commercially cloned into the CRISPR-Cas9 vector (px458, Addgene) from GenScript. The CRISPR plasmids each carrying one of the two different gRNA sequences and a green fluorescence protein (GFP) marker gene were transfected into L3.6pl cells using the Lipofectamine 3000 reagent in serum-free media according to the manufacturer’s guidelines. After 4 h at 37 °C and 5% CO_2_, the serum-free medium was replaced with complete RPMI1640 media. The cells were then observed under the microscope 48 h post transfection, to record the transfection efficiency of L3.6pl cells (via the GFP signal) and to test for any toxicity associated with the reagents. The transfected cells were then GFP sorted as single cells into 96 well plates. These cells were expanded into small colonies (clonal expansion) and the cells were trypsin treated and grown in bigger dishes. In total, eleven colonies were obtained. The cells were further screened by on-target site PCR following genomic DNA extraction using the primers pairs—forward 5′-AGGTGCTTTCCATTGCTCATG-3′ and reverse 5′AGTTCCCTAACGTCTGTTCCC-3′. While many of the expanded clones gave full length (2200 bp), one clone demonstrated a significant deletion resulting in a truncated DNA fragment (750 bp). Subsequent DNA sequencing and cDNA sequencing of this fragment confirmed the deletion of exon 2 and exon 3 sequences within the *ATP13A3* gene and demonstrated that the deletion was in-frame (see Supporting Information).

### MTS assays

Cells were seeded into a 96-well plate at a density of 500 cells/well using 90 µL of complete media and aminoguanidine (250 µM). After 24 h, 10 µL of DFMO stock was added to the well to give a final volume of 100 µL. Plates were incubated for 72 h at 37 °C/5% CO_2_ before adding 20 µL of CellTiter 96 Aqueous One Solution Cell Proliferation Assay (Promega) to each well, followed by another 4 h incubation at 37˚C. Absorbance was recorded at 490 nm using a SynergyMx (BioTek) plate reader. The background (no cells) and untreated controls (with cells) were run in parallel in separate wells containing media (90 µL) + PBS (10 µL). Relative growth was then calculated.

### Radiolabeled polyamine uptake assay

Cells were seeded in 24-well plates at a density of 100,000 cells/mL in complete media with aminoguanidine (250 µM). The next day, the media were replaced with preheated 1X HBSS (Gibco, 270 µL) containing Ca^2+^ and Mg^2+^. Then, ^3^H-Spd, ^3^H-Put or ^14^C-Spm (0.5–3 µM, 30 µL) was added to the wells and plates were incubated at 37 °C for 15 min. In the untreated wells, 0.01 N HCl (30 µL) was added. At the end of incubation, plates were transferred onto ice and washed with cold HBSS (Gibco) three times followed by the addition of 0.1% sodium dodecyl sulfate (SDS, 300 µL). After 10 min, the cell lysate was scraped off the wells and transferred into microcentrifuge tubes, incubated at 85˚C for 10 min, then centrifuged at 15,000 rpm for 10 min. 200 µL of each lysate was transferred to a respective scintillation vial containing scintillation fluid (2 mL of Scintiverse DB, Fisher) and the radioactivity was assessed using a Beckman Coulter LS6500 scintillation counter. The resulting scintillation counts were normalized to the respective protein amount that was quantified using the Pierce BCA protein assay kit and reported as nmol ^3^H-Spd/µg protein, nmol ^3^H-Put/µg protein or ^14^C-Spm/µg protein. The Vmax was then calculated using the Lineweaver–Burk plot and the equation: 1/Vo = (Km/Vmax)1/[S] + 1/Vmax.

### Intracellular polyamine quantification using HPLC

50,000 cells/mL of cells were seeded in RPMI-1640 medium with aminoguanidine (250 µM) in a 10 cm culture dish. After 24 h, the cells were washed three times with cold PBS and trypsinized (0.25% Trypsin- EDTA), collected and washed two times with cold PBS to obtain the respective cell pellets. The cell pellets were then resuspended in 150 µL of perchloric acid (PCA) buffer (0.2 M HClO_4_/1 M NaCl) and 50 µL of aqueous NaCl (0.9%) solution. Cells were homogenized by sonication and the cell suspension was centrifuged at 4000 rpm for 10 min. The resulting supernatant was collected, and the pellets were stored for protein analysis using the BCA kit. The supernatant (100 µL) was transferred into a 4 mL glass vial for polyamine N-dansylation via a modified method of Minocha et al.^28^ Briefly, 10 µL of 1,7-heptanediamine (400 µM in 2% PCA solution) was added to the supernatant and vortexed. Next, 240 µL of aqueous carbonate buffer (pH 9.5) and 400 µL of freshly prepared dansyl chloride solution (20 mg/mL in dry acetone) were added. After vortexing, the samples were incubated at 60 °C for 1 h with mixing at 200 rpm. Further, L-alanine was added and incubated for 15 min at 60 °C to quench the remaining dansyl chloride. Samples were then concentrated to dryness. To this residue, 300 µL of water and 1 mL of chloroform were added, followed by vigorous mixing. The bottom organic layer was separated by pipet and the organic layer concentrated under vacuum to provide a residue which was dissolved in 1 mL of methanol. The respective methanol solution was then filtered through a C18 plug into a fresh vial, followed by an additional rinse through with 500 µL of methanol. The methanol solution (1.5 mL) was then analyzed for intracellular polyamine levels using a Shimadzu High Performance Liquid Chromatograph (HPLC) and the data was normalized over protein and reported as nmol polyamine/mg protein ± standard deviation. All the experimental conditions were performed in triplicate.

### ATP13A3 functional rescue experiments

ATP13A3-FD cells were transfected either with a commercially synthesized plasmid carrying the wild-type ATP13A3 construct fused to FusionRed-MQV tag in a pcDNA3.1 vector backbone (GenScript) or the empty pcDNA3.1 vector using Lipofectamine 3000. A stable cell line was generated using G418 antibiotic selection at a concentration of 250 µg/mL. The transfected cells were seeded on cover slips at a concentration of 40,000–50,000 cells in a 24 well plate with 250 μM aminoguanidine in 500 μL of media per well and incubated overnight at 37 °C/5% CO_2_. For the NBD-NMe-SPM uptake assay using confocal microscopy, the cells were then treated with 1 µM NBD-NMe-SPM for 4 h. The cells were fixed with 4% PFA for 20 min at room temperature. Following washes with PBS, the cells were permeabilized with 0.01% Triton X-100 in PBS for 10 min at room temperature. Coverslips were mounted onto slides using a drop of mounting media with DAPI (Southern Biotech). For examining the localization of the overexpressed ATP13A3 in the ATP3A3-FD/WT rescue cells under DFMO pressure, the transfected cells were seeded on cover slips at a concentration of 40,000–50,000 cells in a 24 well plate with DFMO (4.2 mM) for 72 h. The cells were then fixed and mounted onto slides as described above.

### Analysis of clinical significance of ATP13A3 mRNA and protein expression

Correlation of ATP13A3 mRNA expression with genes of the polyamine metabolic pathway was performed using the cBioPortal (https://www.cbioportal.org)^29^. Using the “Pancreatic Adenocarcinoma (TCGA, PanCancer Atlas)” dataset within the cBioPortal, we identified the inverse or positive correlation between ATP13A3 mRNA expression and SRM, PAOX, OAZ1, or CDK6 with the help of the cBioPortal “Co-expression” tool. Data were downloaded from the cBioPortal and for further analysis Prism9 was used. To express the level of correlation, we used the Pearson correlation coefficient. To identify ATP13A3’s correlation with pancreatic cancer patient survival, we used within the UCSC Xena Browser (https://xenabrowser.net)^30^ the “GDC TCGA Pancreatic Cancer” dataset. Kaplan Meier plot data were downloaded from the Xena Browser and only primary adenocarcinoma data and data from uninvolved normal tissue were used for further analysis. mRNA expression data for ATP13A3 and SRM were used for Kaplan Meier analysis in Prism with log2 expression values for ATP13A3 > 17.6 and SRM log2 expression > 19.25 regarded as “high”. To determine statistical significance, we used the Log-rank (Mantel-Cox) test. Single cell ATP13A3 protein expression data (fluorescence intensities) from at least 20 KRT8 + normal and tumor cells for each of the 4 PDAC patients were used and normal versus tumor was evaluated using an unpaired Student’s t test.

### Tissue Microarray Array (TMA) immunofluorescence staining

Tissue slides were treated as previously described^31^. Primary antibodies against KRT8 (1:200; Santa Cruz Biotechnology, clone Ks8-7) and rabbit ATP13A3 (1:400) were applied in 1X PBS/1%BSA solution and incubated overnight at room temperature in a humidified chamber. Slides were then washed three times with 1X PBS and further incubated with secondary antibodies (anti-mouse Alexa 633, anti-rabbit biotinylated at 1:200) for 20 min in at room temperature. Another additional step was performed with anti-neutravidin 568 (for biotinylated antibody) at 1:200 for 20 min. Slides were finally washed thrice with 1X PBS and coverslips mounted with DAPI mounting medium. Random fields were imaged with a Leica SP5 confocal microscope (Leica Microsystems Inc, Buffalo Grove, IL, USA) using the 63X objective. Representative images are shown, and the white scale bar applies to each slide and all images were taken at the same magnification.

### Statistical analysis

All statistical analyses were performed using unpaired *t* test, Student’s *t* test, one-way ANOVA or 2-way ANOVA for independent measures using GraphPad Prism.

### Material transfer agreement

A Material Transfer Agreement was executed by UCF in order to obtain the original L3.6pl cells from Dr. Isaiah Fidler at MD Anderson Cancer Center (MDACC) in Houston, TX. This agreement included a consent to use these metastatic cells for research purposes. The original study at the MD Anderson Cancer Center to isolate these cells from mice received ethical approval from the MDACC IACUC committee.

## Supplementary Information

Supplementary Information.
