# Supplementary material for: ATP13A3 facilitates polyamine transport in human pancreatic cancer cells
Source: Sci Rep. 2022 Mar 8;12:4045. doi: 10.1038/s41598-022-07712-4 (PMC8904813; doi:10.1038/s41598-022-07712-4)
Supplement: Supplementary file 1 — Supplementary Information. [file 41598_2022_7712_MOESM1_ESM.docx]

**Supplementary Information**

ATP13A3 facilitates polyamine transport in human pancreatic cancer cells

Vandana Sekhar, Thomas Andl, and Otto Phanstiel IV*

* Otto Phanstiel IV

**Email:** [otto.phanstiel@ucf.edu](mailto:otto.phanstiel@ucf.edu)

**Table of Contents page(s)**

**Figure SI 1**. Increased expression of the full-length ATP13A3 protein in AsPC-1 S3

cells exposed to polyamine stress

**Figure SI 2**. SLC12A8 does not play a role in polyamine transport in pancreatic S4-5

cancer cells

**Figure SI 3. (a)** The coordinates on the chromosome 3 in the human genome S6

sequence showing where the gRNA sequences targeted the *ATP13A3* gene

along with sequence of the gRNA. **(b)** ATP13A3-FD Protein sequence with

a 51 amino acid deletion (1175 aa).

**Figure SI 4** Graphical representation of the intracellular levels of putrescine, S7

spermidine, spermine and the total polyamines (PA) in the wild type and

ATP13A3-FD as detected via N-dansylation and by HPLC.

**Figure SI 5.** Graphical representation of the decreased growth of ATP13A3-FD S8

L3.6pl cells in response to increasing doses of DFMO for 72h.

**Figure SI 6.** Relative expression of the SLC12A8 protein in L3.6pl WT and S9

ATP13A3-FD cells.

**Figure SI 7.** Clinical significance of ATP13A3 and its expression in PDAC S10-12

Figure SI 8. Original Western blot images S13-15


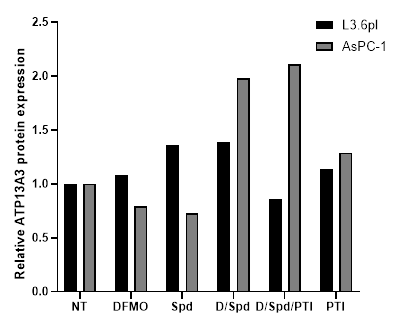


**Fig. SI 1 Increased expression of the full-length ATP13A3 protein in AsPC-1 cells exposed to polyamine stress.** Quantification of the relative expression of full-length ATP13A3 (band marked with *) in L3.6pl and AsPC-1 cells following treatment with different polyamine stimuli normalized to β-actin loading control.

(a) (b)


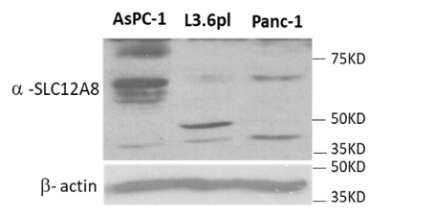

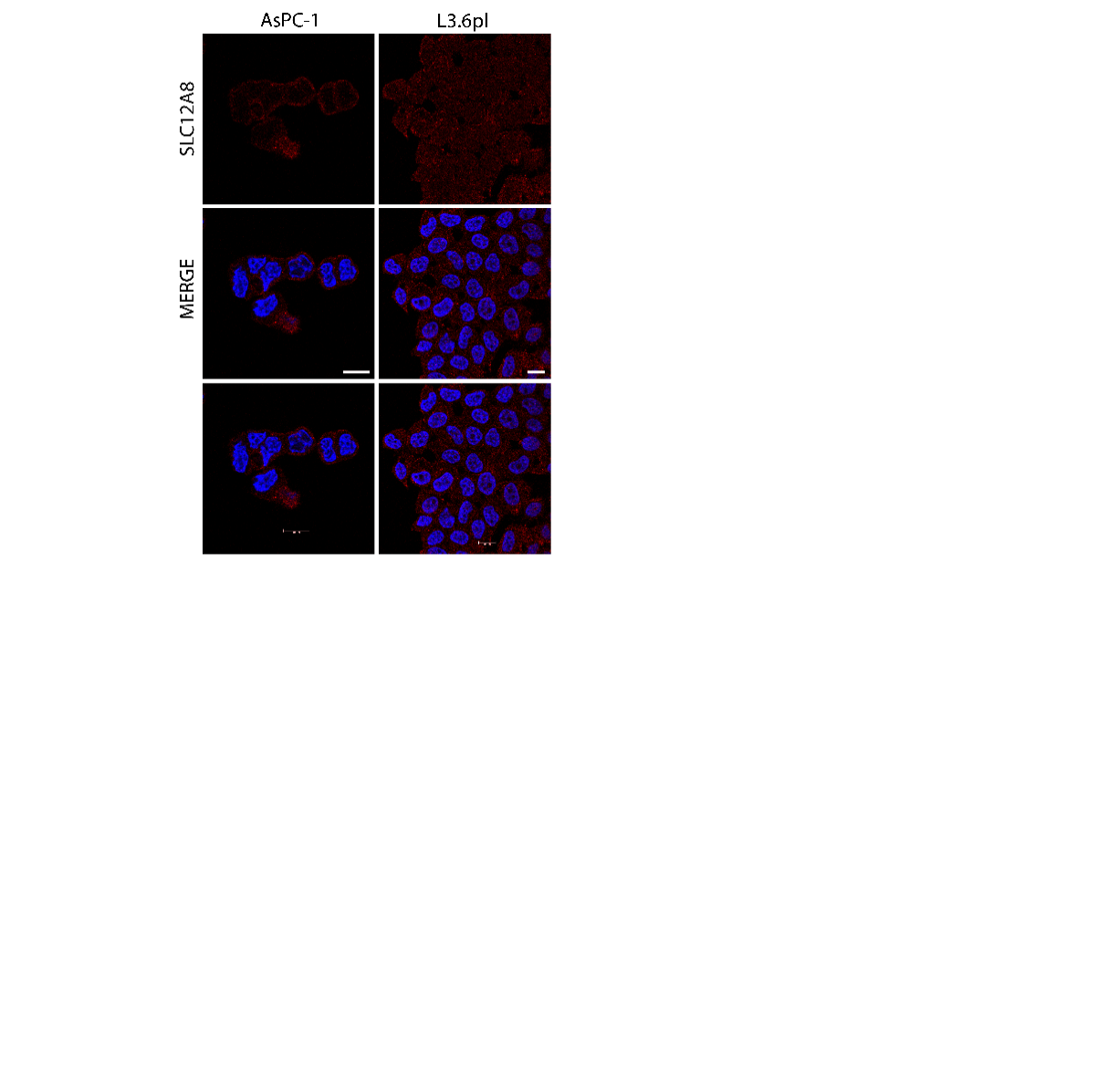


(c)


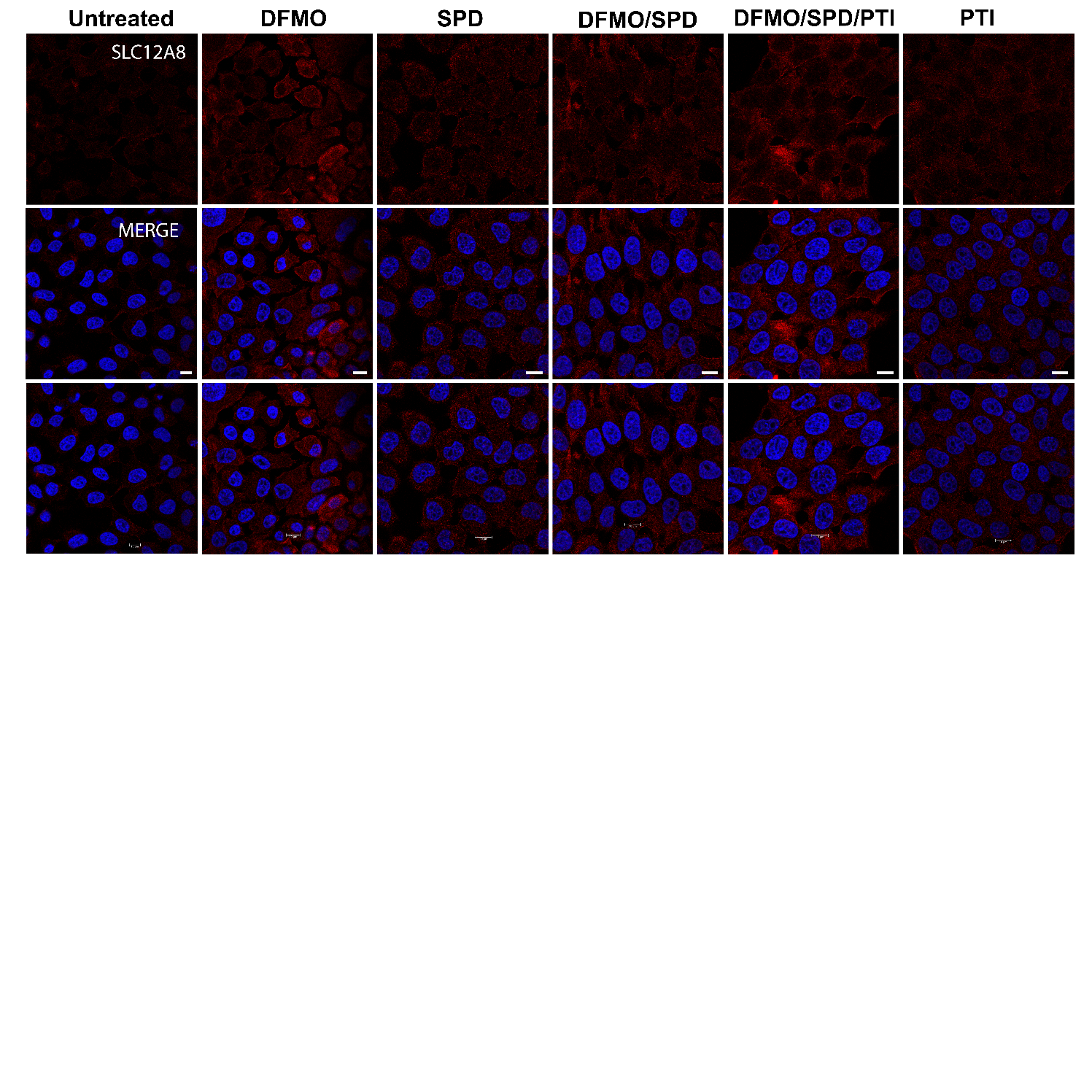


(d)


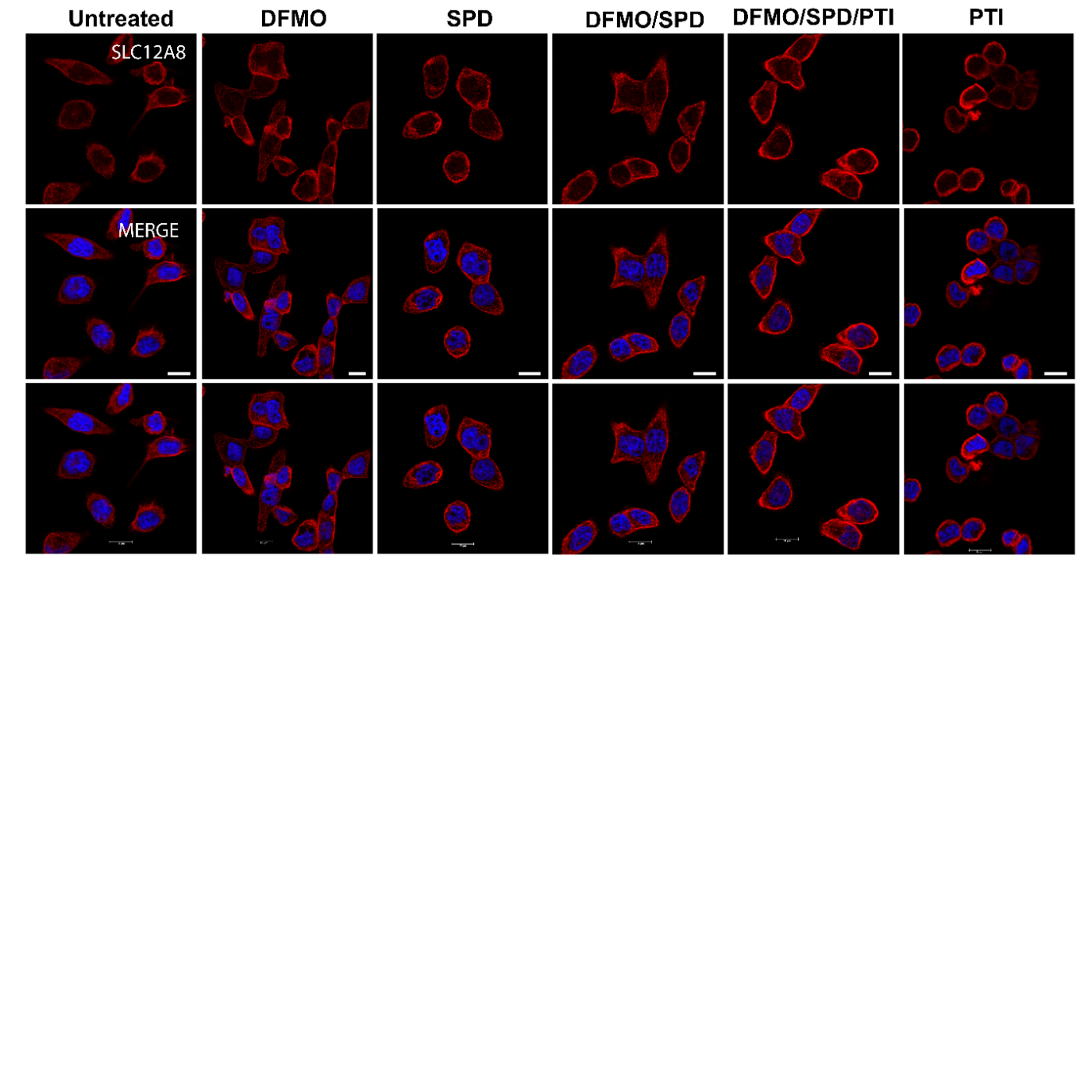


Fig. SI 2. SLC12A8 does not play a role in polyamine transport in pancreatic cancer cells (a) Expression of the SLC12A8 protein in AsPC-1, L3.6pl, and Panc-1 cells. β-actin was used as a loading control. (b) Confocal microscopy images showing the cellular localization of SLC12A8 in the AsPC-1 and L3.6pl. SLC12A8 is shown in red and the DAPI stained nuclei is shown in blue in the merged image. Confocal microscopy images of L3.6pl cells (c) and AsPC-1 cells (d) treated with DFMO, Spd, DFMO/Spd, DFMO/Spd/PTI or PTI only. ATP13A3 protein is shown in green, SLC12A8 in red and DAPI stained nuclei in blue can be seen in the merged images. (Scale bar, 10 µm).

**(a)**

The G is at position 194462185 on chromosome 3 in the human hg38 version (**UCSC Genome Browser on Human Dec. 2013 (GRCh38/hg38) Assembly)**. The T is at position 194460726. 1458bp are deleted using Crispr/Cas9 with two sgRNAs: one sgRNA in Exon2 and one in Exon3 of the ATP13A3 gene (RefSeq: NM_024524.4, i.e., ATP13A3 transcript variant 1). The gRNA sequences are underlined and cursive, the PAM site in bold. Cas9 cut on both sides exactly 3 nucleotides upstream of the PAM site.

EXON2-TT*AATCCACTGTAGGTATGGAC***AGG**-1458bp deletion- **CCT***GAGTGGCGGGTGAAAGCGAC*CT-EXON3

**(b)**

M*DELETION*WRVKATCVRAAIKDCEVVLLRTTDEFKMWFCAKIRVLSLETYPVSSPKSMSNKLSNGHAVCLIENPTEENRHRISKYSQTESQQIRYFTHHSVKYFWNDTIHNFDFLKGLDEGVSCTSIYEKHSAGLTKGMHAYRKLLYGVNEIAVKVPSVFKLLIKEVLNPFYIFQLFSVILWSTDEYYYYALAIVVMSIVSIVSSLYSIRKQYVMLHDMVATHSTVRVSVCRVNEEIEEIFSTDLVPGDVMVIPLNGTIMPCDAVLINGTCIVNESMLTGESVPVTKTNLPNPSVDVKGIGDELYNPETHKRHTLFCGTTVIQTRFYTGELVKAIVVRTGFSTSKGQLVRSILYPKPTDFKLYRDAYLFLLCLVAVAGIGFIYTIINSILNEVQVGVIIIESLDIITITVPPALPAAMTAGIVYAQRRLKKIGIFCISPQRINICGQLNLVCFDKTGTLTEDGLDLWGIQRVENARFLSPEENVCNEMLVKSQFVACMATCHSLTKIEGVLSGDPLDLKMFEAIGWILEEATEEETALHNRIMPTVVRPPKQLLPESTPAGNQEMELFELPATYEIGIVRQFPFSSALQRMSVVARVLGDRKMDAYMKGAPEAIAGLCKPETVPVDFQNVLEDFTKQGFRVIALAHRKLESKLTWHKVQNISRDAIENNMDFMGLIIMQNKLKQETPAVLEDLHKANIRTVMVTGDSMLTAVSVARDCGMILPQDKVIIAEALPPKDGKVAKINWHYADSLTQCSHPSAIDPEAIPVKLVHDSLEDLQMTRYHFAMNGKSFSVILEHFQDLVPKLMLHGTVFARMAPDQKTQLIEALQNVDYFVGMCGDGANDCGALKRAHGGISLSELEASVASPFTSKTPSISCVPNLIREGRAALITSFCVFKFMALYSIIQYFSVTLLYSILSNLGDFQFLFIDLAIILVVVFTMSLNPAWKELVAQRPPSGLISGALLFSVLSQIIICIGFQSLGFFWVKQQPWYEVWHPKSDACNTTGSGFWNSSHVDNETELDEHNIQNYENTTVFFISSFQYLIVAIAFSKGKPFRQPCYKNYFFVFSVIFLYIFILFIMLYPVASVDQVLQIVCVPYQWRVTMLIIVLVNAFVSITVEESVDRWGKCCLPWALGCRKKTPKAKYMYLAQELLVDPEWPPKPQTTTEAKALVKENGSCQIITIT

**Fig. SI 3. (a)** The coordinates on the chromosome 3 in the human genome sequence showing where the gRNA sequences targeted the *ATP13A3* gene along with sequence of the gRNA. **(b)** ATP13A3-FD Protein sequence with a 51 amino acid deletion (1175 aa).

**Fig. SI 4.** Graphical representation of the intracellular levels of putrescine, spermidine, spermine and the total polyamines (PA) in the wild type and ATP13A3-FD as detected via N-dansylation and by HPLC. The polyamines are shown as nmoles PA/mg protein. The data are representative of mean +/- SD of three independent experiments. (****P < 0.0001 and ***P<0. 0001)


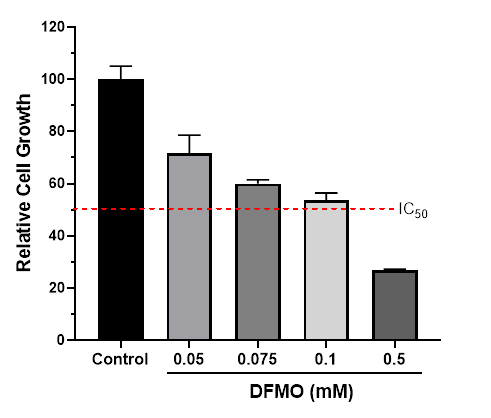


**Fig. SI 5.** Graphical representation of the decreased growth of ATP13A3-FD L3.6pl cells in response to increasing doses of DFMO for 72h. The red line represents the DFMO IC50 value of 0.1 mM.


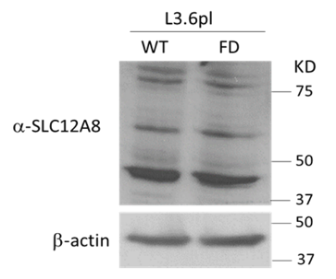


Fig. SI 6. Relative expression of the SLC12A8 protein in L3.6pl WT and ATP13A3-FD cells. β-actin was used as a loading control.


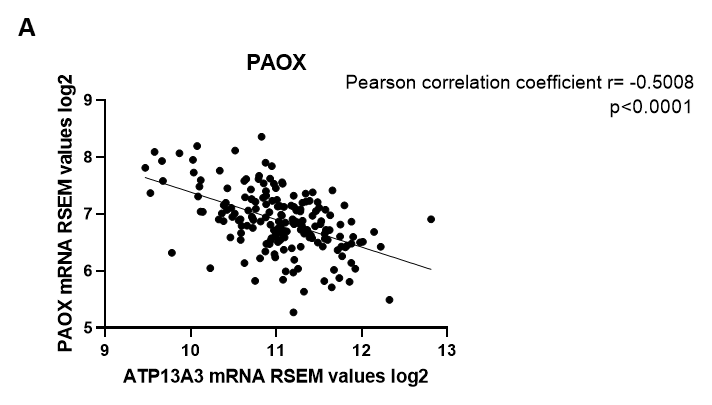


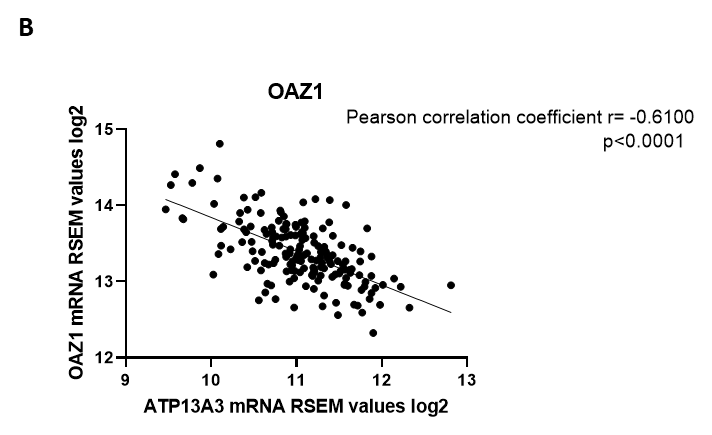


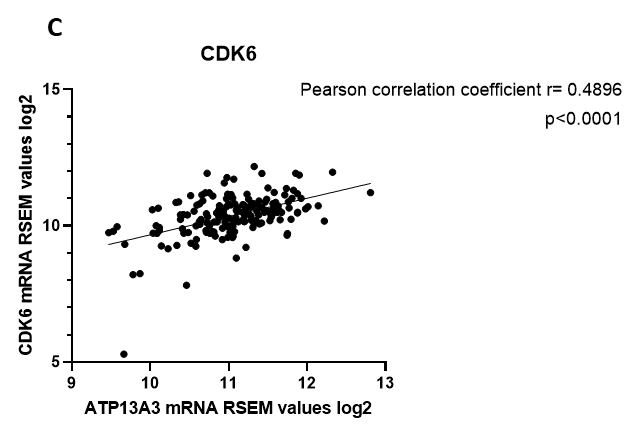


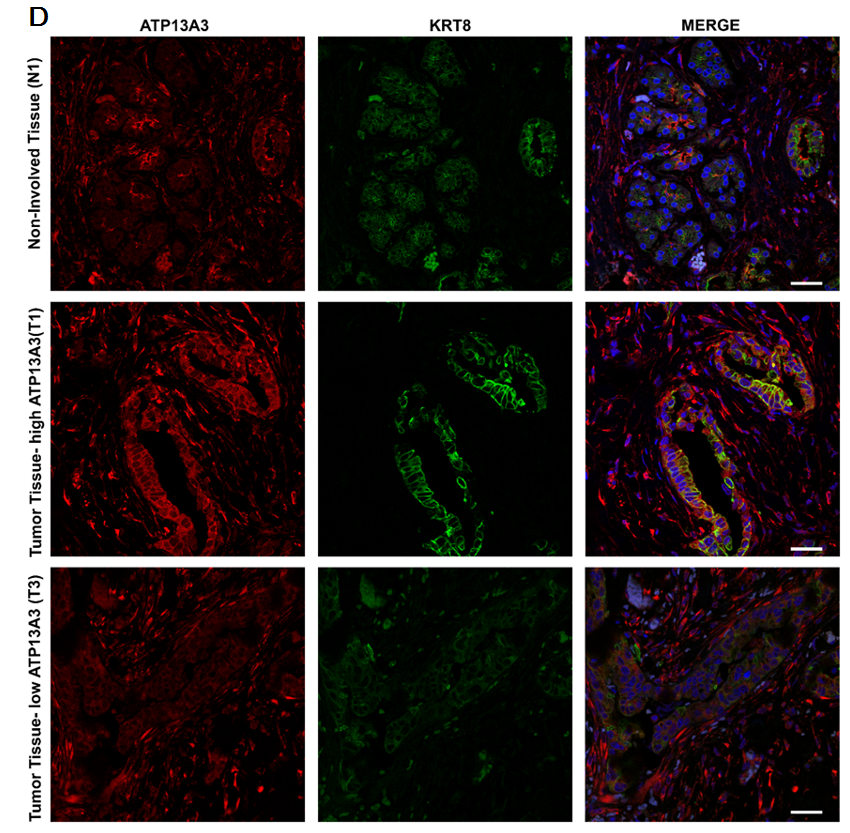


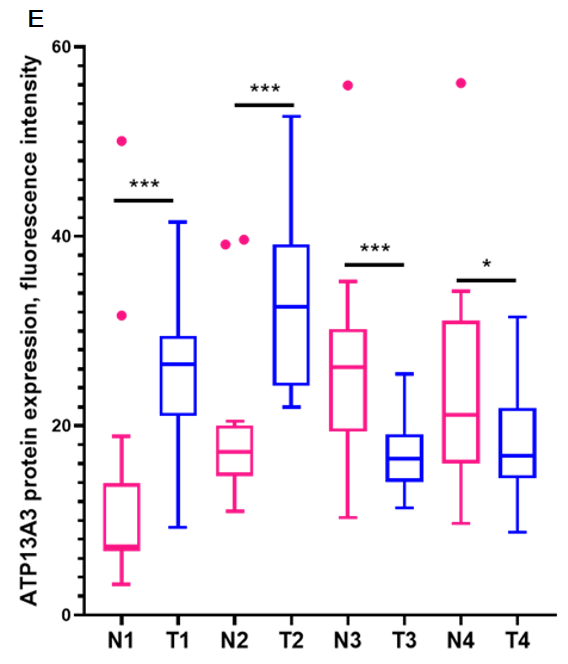


**Fig. SI 7. Clinical significance of ATP13A3 and its expression in PDAC** (a) Graph representing the correlation of PAOX **(A)**, OAZI **(B)** and CDK6 **(C)** mRNA expression with ATP13A3 mRNA expression, obtained using the Pancreatic adenocarcinoma (TCGA, PanCancer Atlas) dataset. **(D)** Representative images showing the expression of ATP13A3 (red) and KRT8 (green) in normal and tumor tissues of PDAC patients. DAPI stained nuclei are shown in blue in the merged images. **(E)** Analysis of single cell ATP13A3 protein expression data (fluorescence intensities) from at least 20 KRT8+ normal and tumor cells for each of the four PDAC patients wherein normal (N) versus tumor (T). Statistical significance was evaluated using an unpaired Student’s t test. (p*** <0.0001, p*=0.0145). Note: the ATP13A3 antibody (Sigma, HPA029471) used may also detect some truncated variants of ATP13A3.

Fig. SI 8. Original Western blot images for each Figure listed:

Fig 1a.


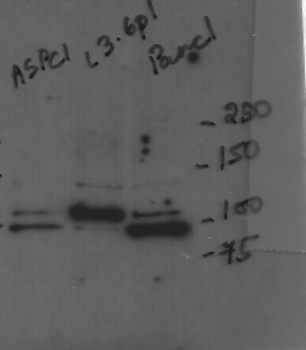

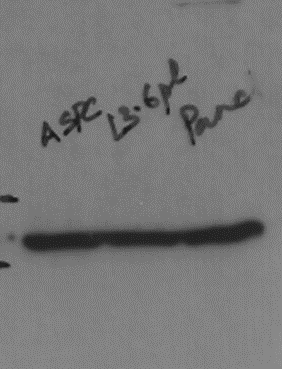


ATP13A3 protein β-actin protein

Fig 2c.


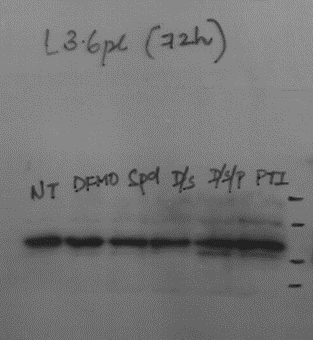

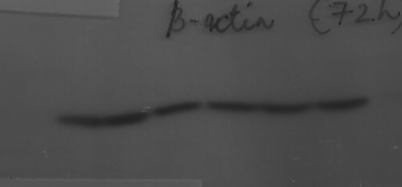


ATP13A3 expression in L3.6pl cells β-actin expression in L3.6pl cells


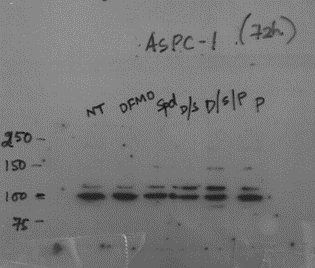

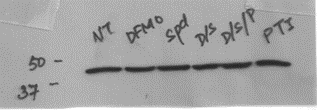


ATP13A3 expression in AsPC-1 cells β-actin expression in AsPC-1 cells

Fig SI 2a.


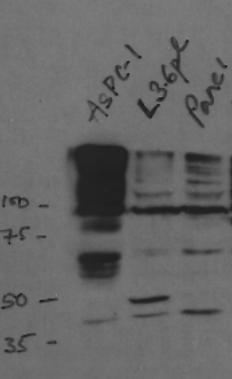

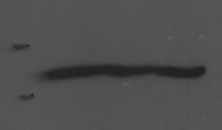


SLC12A8 protein β-actin

Fig SI 6.


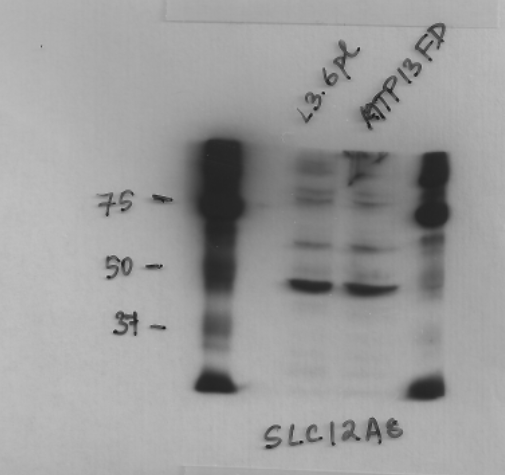

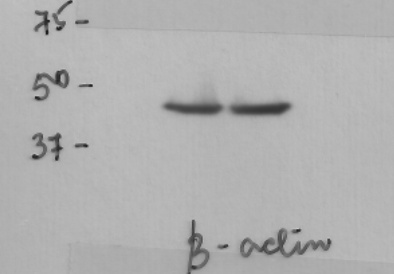


SLC12A8 protein β-actin protein
